# Integrated mRNA- and miRNA-sequencing analyses unveil the underlying mechanism of tobacco pollutant-induced developmental toxicity in zebrafish embryos

**DOI:** 10.1186/s12967-024-05050-9

**Published:** 2024-03-08

**Authors:** Jiasheng Chen, Yuxin Lin, Deyi Gen, Wanxian Chen, Rui Han, Hao Li, Shijie Tang, Shukai Zheng, Xiaoping Zhong

**Affiliations:** Department of Burns and Plastic Surgery, The Second Affiliated Hospital of Shantou University Medical College, Shantou, 515041 Guangdong People’s Republic of China

**Keywords:** Cigarette smoke extract, RNA-seq, Apoptosis disorder, Lipid metabolism

## Abstract

Tobacco pollutants are prevalent in the environment, leading to inadvertent exposure of pregnant females. Studies of these pollutants' toxic effects on embryonic development have not fully elucidated the potential underlying mechanisms. Therefore, in this study, we aimed to investigate the developmental toxicity induced by cigarette smoke extract (CSE) at concentrations of 0.25, 1, and 2.5% using a zebrafish embryo toxicity test and integrated transcriptomic analysis of microRNA (miRNA) and messenger RNA (mRNA). The findings revealed that CSE caused developmental toxicity, including increased mortality and decreased incubation rate, in a dose-dependent manner. Moreover, CSE induced malformations and apoptosis, specifically in the head and heart of zebrafish larvae. We used mRNA and miRNA sequencing analyses to compare changes in the expression of genes and miRNAs in zebrafish larvae. The bioinformatics analysis indicates that the mechanism underlying CSE-induced developmental toxicity was associated with compromised genetic material damage repair, deregulated apoptosis, and disturbed lipid metabolism. The enrichment analysis and RT-qPCR show that the *ctsba* gene plays a crucial function in embryo developmental apoptosis, and the *fads2* gene mainly regulates lipid metabolic toxicity. The results of this study improve the understanding of CSE-induced developmental toxicity in zebrafish embryos and contribute insights into the formulation of novel preventive strategies against tobacco pollutants during early embryonic development.

**Supplementary Information:**

The online version contains supplementary material available at 10.1186/s12967-024-05050-9.

## Introduction

Tobacco consumption remains prevalent, fueled by the growth of the nicotine-addicted population and smokers in low- and middle-income countries [1]. Combustible or non-combustible tobacco products, such as conventional cigarettes and e-cigarettes, produce smoke and aerosol components, respectively. Gas components produced by tobacco, including nicotine, nitrosamines, carbonyls, aromatic volatile organic compounds, and trace metals, share similarities and can pose risks to human health [2, 3]. Furthermore, tobacco smoke, even when dissipated, continuously accumulates on object surfaces, dust, and air, creating potential exposure routes through inhalation, ingestion, and skin transfer [4]. Tobacco pollutants can also permeate nonsmoking areas through shared spaces [5]. Even long after tobacco smoke dissipates, tobacco pollutants remain at high levels, with a potential for an increase in concentrations [6]. Therefore, the universality and persistence of tobacco pollutants lead to the inadvertent exposure of individuals within their living environments.

As age increases and fertility rates decrease, pregnant females prioritize preventing pregnancy-related risks to develop healthy children. Research indicates that tobacco pollutant exposure remains a significant factor in embryonic dysplasia during pregnancy [7]. This exposure increases the incidence of adverse embryonic developmental events such as low birth weight, head and facial malformations, heart defects, and embryonic death [8–10]. The zebrafish pattern is often used to evaluate the correlation between toxic material exposure and embryonic development toxicity [11]. Moreover, the zebrafish pattern is particularly suitable for studying tobacco pollutant-induced developmental toxicity because of its low cost and direct observability of embryonic development processes [12]. Although some studies on tobacco pollutant exposure have focused on the developmental toxicity induced by a single component of a tobacco pollutant mixture, such as nicotine [13, 14], tobacco smoke is a mixture of over 9500 chemical components [15]. Therefore, studies exclusively examining a single component do not represent the global effects of tobacco pollutant exposure on embryos [16]. Moreover, most studies using a tobacco pollutant mixture have focused on visible embryonic developmental toxicity, such as malformations, with a limited exploration of the specific molecular mechanisms involved in these processes [17, 18]. Therefore, studying possible underlying molecular mechanisms and the overall effects of tobacco smoke exposure on embryonic development can overcome the limitations of existing studies and promote the development of new and effective preventive measures during pregnancy.

High-throughput sequencing technologies, such as messenger RNA (mRNA) or microRNA (miRNA) sequencing, provide effective tools for studying global transcriptomic changes and mechanisms in zebrafish embryo-larvae in response to harmful external stimuli [19, 20]. Recently, changes in the mRNA transcriptome during developmental toxicity in zebrafish have been described [21, 22]. miRNA, which controls the expression of mRNAs by inhibiting translation or directly grading transcription processes, plays a fundamental role in response to symbiotic stress, which is essential for toxicology research and is gradually gaining attention from researchers [23, 24]. However, the change in miRNA and mRNA expression profiles in response to tobacco smoke stress during the early embryonic development of zebrafish has not been described. Therefore, conducting a detailed comparative transcriptome analysis of miRNA and mRNA expression in zebrafish embryos exposed to varying concentrations of tobacco is essential to deepen the understanding of the underlying mechanism of tobacco pollutant-induced developmental toxicity.

In this study, we aimed to improve the understanding of the effects of tobacco exposure on zebrafish embryonic development. Thus, we treated zebrafish embryos with different concentrations of cigarette smoke extract (CSE) to assess developmental toxicity. Sequencing technology was used to compare the expression profiles of miRNAs and mRNAs in zebrafish larvae, followed by comprehensive analysis. Subsequently, the expression of crucial genes was determined using quantitative real-time PCR (RT-qPCR). The results of embryotoxicity tests and bioinformatics analyses improve the understanding of the potential mechanisms underlying developmental toxicity induced by cigarette smoke and provide a theoretical basis for formulating effective measures against tobacco pollutants during pregnancy.

## Materials and methods

### Chemicals and reagents

Using a previously described protocol [25], CSE was freshly prepared from filtered cigarettes within 30 min of use. The acquired CSE suspension was yellowish with an optical density of 0.506 ± 0.008 at 405 nm. The CSE was adjusted to a pH value of 7.4 and filtered using a 0.22 μm filter. The final product was considered to have a concentration of 100% and was diluted to the desired concentrations for further experiments. TRIzol reagent was purchased from Tiangen (Nanjing, China). The PrimeScript RT reagent kit with gDNA Eraser was purchased from Thermo Scientific (MD, USA). The SYBR Green I kit was purchased from Takara Bio Inc. (Shiga, Japan). The other reagents used in this study were purchased from Beyotime Biotechnology (Jiangsu, China).

### Zebrafish maintenance and CSE exposure

Adult zebrafish (wild-type AB strain, *Danio rerio*) were used as research subjects. Zebrafish were maintained under a standard temperature of 28 ± 1 ℃, pH 7.2 ± 2.0, with a lighting cycle of 14 h of light and 10 h of darkness daily. The zebrafish were fed hatched brine shrimp and dry flake food (Nutreco Skretting) twice daily. To obtain fresh embryos, we placed healthy and sexually mature zebrafish in a feeding box at female-to-male ratios of 1:1 or 1:2 for mating and spawning. Animal experiments were approved by the Animal Ethics Committee of Shantou University Medical College (Approval No.: SUMC2022-062).

Two hours post fertilization (hpf), normally developed embryos were selected and randomly distributed in a 10 cm petri dish and treated with corresponding concentrations of CSE (0, 0.25, 1, and 2.5%). CSE exposure concentrations were selected according to the lethal concentration 50 and effective concentration 50 values of embryonic zebrafish. After screening for death and deformity, the embryos which placed in 96-well plates (50 embryos per group) at 48 hpf were transferred and continue incubated in 10 cm Petri dishes for subsequent CSE exposure experiments up to 7 days post fertilization (dpf). All experiments were repeated thrice.

### Effects of CSE on survival and developmental status of zebrafish

The number of dead zebrafish embryos at different concentrations was recorded every 24 h using a stereomicroscope (SZX7, Olympus, Tokyo, Japan). The heart rate (beats/min) of 10 larvae from each group was measured using a stereomicroscope at 96 hpf and recorded using a stopwatch for 1 min. The number of hatched and deformed embryos was recorded to calculate the percentage of larval survival, larvae hatched, and larval malformation (all at 96 hpf).

The developmental conditions of 10 randomly selected zebrafish from each group were observed at 72 hpf and 7 dpf. The larvae were anesthetized using tricaine, and morphological changes were captured and recorded using a stereomicroscope. Specifically, observations focused on developmental indicators, such as morphological changes in the head, presence of pericardial edema, delayed yolk sac absorption, spinal curvature, uneven distribution of pigmentation, development of swim bladders, and shortening of body length.

### Acridine orange staining

Acridine orange (AO) staining of 96-hpf larvae was performed to detect cell apoptosis. In brief, larvae were washed with phosphate buffer saline twice, incubated with 5 mg/mL AO at 28 ℃ in the dark for 20 min, and washed thoroughly with phosphate buffer saline thrice, as described in previously [26]. Images of the zebrafish embryos were captured using an inverted fluorescence microscope (Zeiss, USA) and processed using ImageJ software (Ver. 1.52a; NIH, Bethesda, MD, USA).

### Global transcriptome sequencing (mRNA- and miRNA-seq) analysis

At 7 dpf, the larvae were sacrificed using an overdose of a buffered anesthetic solution of tricaine methanesulfonate (0.03% MS-222; Sigma-Aldrich, USA). Larvae (15–20/pool) were collected, flash-frozen in liquid nitrogen, and stored at − 80 ℃ before molecular analyses. TRIzol reagent was used to isolate total RNA from each sample. High-throughput full transcriptome and miRNA sequencing analyses were performed at Applied Protein Technology Co., Ltd. (Shanghai, China).

### Integrated bioinformatics analyses of the sequencing results

The expression value of miRNA- and mRNA-seq in reads per million and fragments per kilobase of exon model per million mapped fragments, respectively, were acquired after cleansing, mapping, and quantitative analysis of the raw sequencing results, which were completed by Applied Protein Technology Co., Ltd., China. The R packages DESeq and edgeR were used for differential expression analysis of mRNAs and miRNAs between the control and CSE-exposed treatments, respectively. Differentially expressed genes (DEGs) and miRNAs (DEMs) were identified using an adjusted p value (padj) or a false discovery rate < 0.05 and |log2(fold change)|> 1. Subsequently, the DEGs of each treatment were uploaded to STRING (http://string-db.org/) to determine their interaction, which was visualized using Cytoscape software. The hub genes in the protein–protein interaction network with the highest Molecular Complex Detection score were obtained using the Cytoscape plug-in CytoHubba. Finally, DEM target genes were predicted from the miRWalk database (http://mirwalk.umm.uni-heidelberg.de).

Functional enrichment analyses of the Kyoto Encyclopedia of Genes and Genomes (KEGG) and GeneOntology (GO) were conducted for three datasets with different concentrations of CSE-regulated DEGs and hub genes. KEGG and GO enrichment results with a p-value of < 0.05 were considered statistically significant. Networks were established to reveal the relationship between enriched genes and the significant functional enrichment results of the DEGs. In addition, gene set enrichment analysis (GSEA) was conducted for three datasets with different concentrations of CSE-regulated DEGs and DEM target genes. The screening conditions for GSEA were as follows: |normalized enrichment score (NES)|> 1, padj < 0.25, and p value < 0.05. To explore the potential relationship between miRNAs and mRNAs, we first crossed the significant GSEA-KEGG results of DEM target genes and all CSE-regulated DEGs to obtain the candidate KEGG pathway and constructed miRNA–mRNA–pathway regulatory networks with miRNA as a decoy, mRNA as a center, and pathway as a target. These networks were visualized using Cytoscape software. We used the intersection of significant GO terms between miRNA and mRNA levels to demonstrate the GO results by using upset and dot plots.

### RT-qPCR for verification

Total RNA was extracted using TRIzol reagent and subjected to complementary DNA (cDNA) synthesis using the RevertAid First Strand cDNA Synthesis Kit. RT-qPCR was performed using SYBR Green I according to the manufacturer’s instructions. The primer sequences are listed in Additional file 1: Table S1.

### Statistical analysis

Data are expressed as mean ± standard deviation, obtained from experiments with three replicates of different CSE concentrations. One-way analysis of variance with Dunnett's post hoc test was used to analyze the effects of CSE exposure on developmental toxicity. Differences were considered statistically significant at p < 0.05. SPSS software (version 27.0; SPSS, Chicago, IL, USA) was used for statistical analyses.

## Results

### Embryonic development toxicity of CSE

**Fig. 1:**
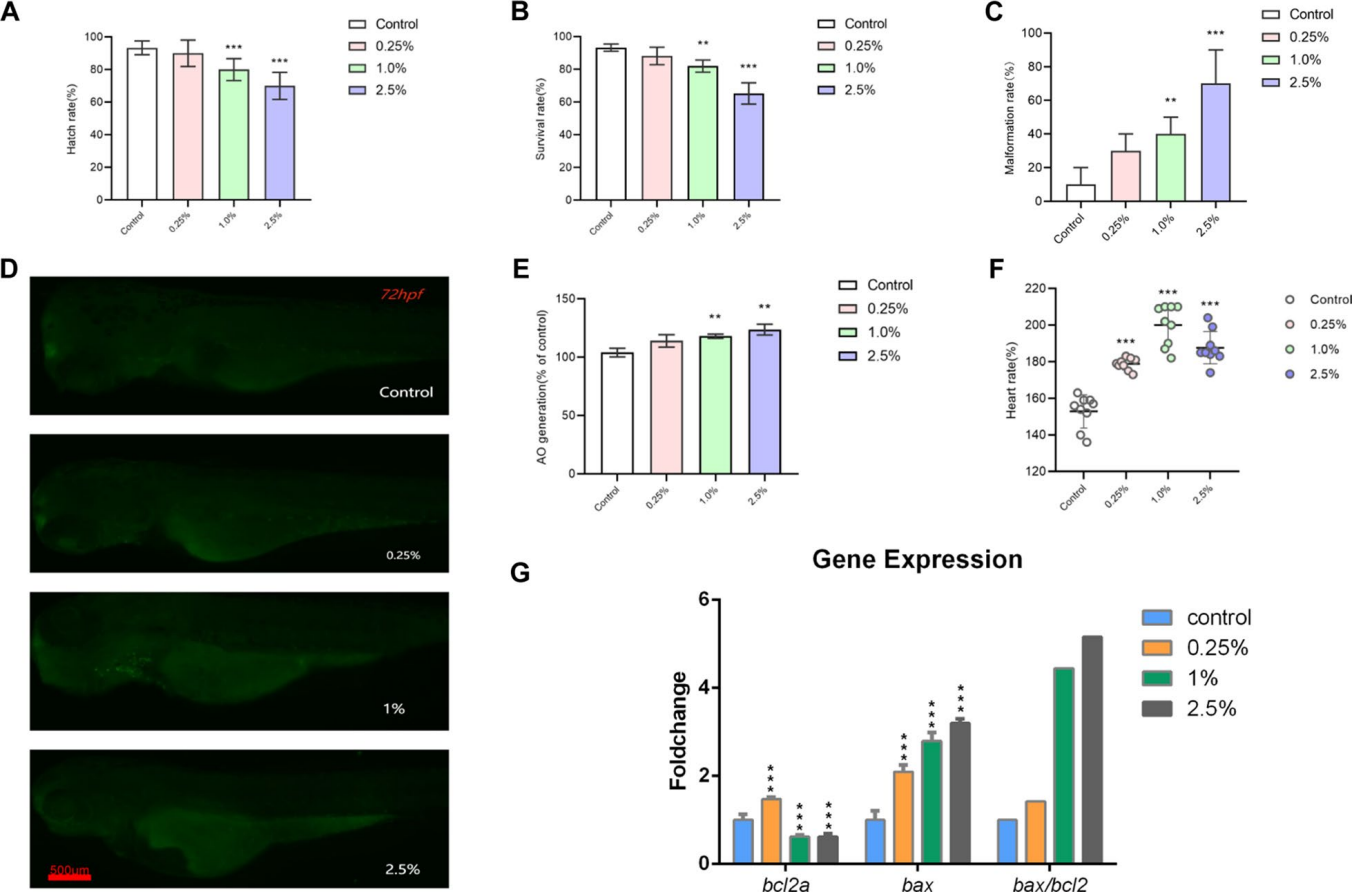
Toxic effects of CSE on the development of zebrafish embryos. The hatching rate (**A**), survival rate (**B**) and malformation rate (**C**) of zebrafish larvae exposed to various concentrations of CSE (0, 0.25, 1 and 2.5%) for 96 hpf. Lateral view (**D**) and the relative fluorescence intensities (**E**) of bright spots region in control and CES-exposure groups in 96-hpf larvae after AO staining. The heart rate of zebrafish larvae exposed to various concentrations of CSE (0, 0.25, 1 and 2.5%) for 96 hpf (**F**). Expression of apoptosis-related genes (Bcl-2, Bax, Bax/Bcl-2) in the 96-hpf zebrafish larvae after CSE exposure by qRT-PCR (**G**). Results were presented as the mean ± SD of three independent experiments. Each group included 3 replicates (10 larvae/replicate). *P < 0.05, **P < 0.01, ***P < 0.001 compared with the control group

The treatment groups exhibited lower hatching and survival rates and higher mortality rates than the control group, particularly evident in the 2.5% CSE treatment group at 96 hpf (Fig. 1A–C). The results demonstrated a dose-dependent effect on zebrafish embryos exposed to CSE (0.25, 1, and 2.5%) compared to the control group. In addition, the heart rates in the 0.25, 1, and 2.5% CSE groups were higher than those in the control group at 96 hpf (Fig. 1F).

Typical malformations, including morphological changes in the head, presence of pericardial edema, delayed yolk sac absorption, spinal curvature, uneven distribution of pigmentation, absence of swim bladders, and shortening of body length, were observed at 72 hpf and 7 dpf and are presented in Additional file 2: Fig. S1.

### Effects of CSE exposure on larvae injury and cell apoptosis

AO staining assay was conducted to determine the occurrence of cell apoptosis after CSE exposure. At 72 hpf, no distinct apoptotic cells were observed in the control group. However, in the CSE-exposed treatment groups, apoptotic cells primarily occurred in the head, heart, and surrounding tissues, indicating notable concentration dependence (Fig. 1D). The *bax*/*bcl-2* genes serve as markers of apoptosis, and an imbalance in their expression plays an important role in apoptosis [27]. To further investigate the mechanisms underlying CSE-induced apoptosis, we assessed the expression level of *bax*/*bcl-2*. CSE exposure significantly decreased the expression of *bcl-2* mRNA (anti-apoptotic gene) but increased that of *bax* (pro-apoptotic gene) in larval zebrafish (Fig. 1G). These results indicate a strong correlation between cell apoptosis and the injury observed in zebrafish larvae after CSE exposure.

### Changes in transcriptome profile of miRNA and mRNA after CSE exposure

**Fig. 2:**
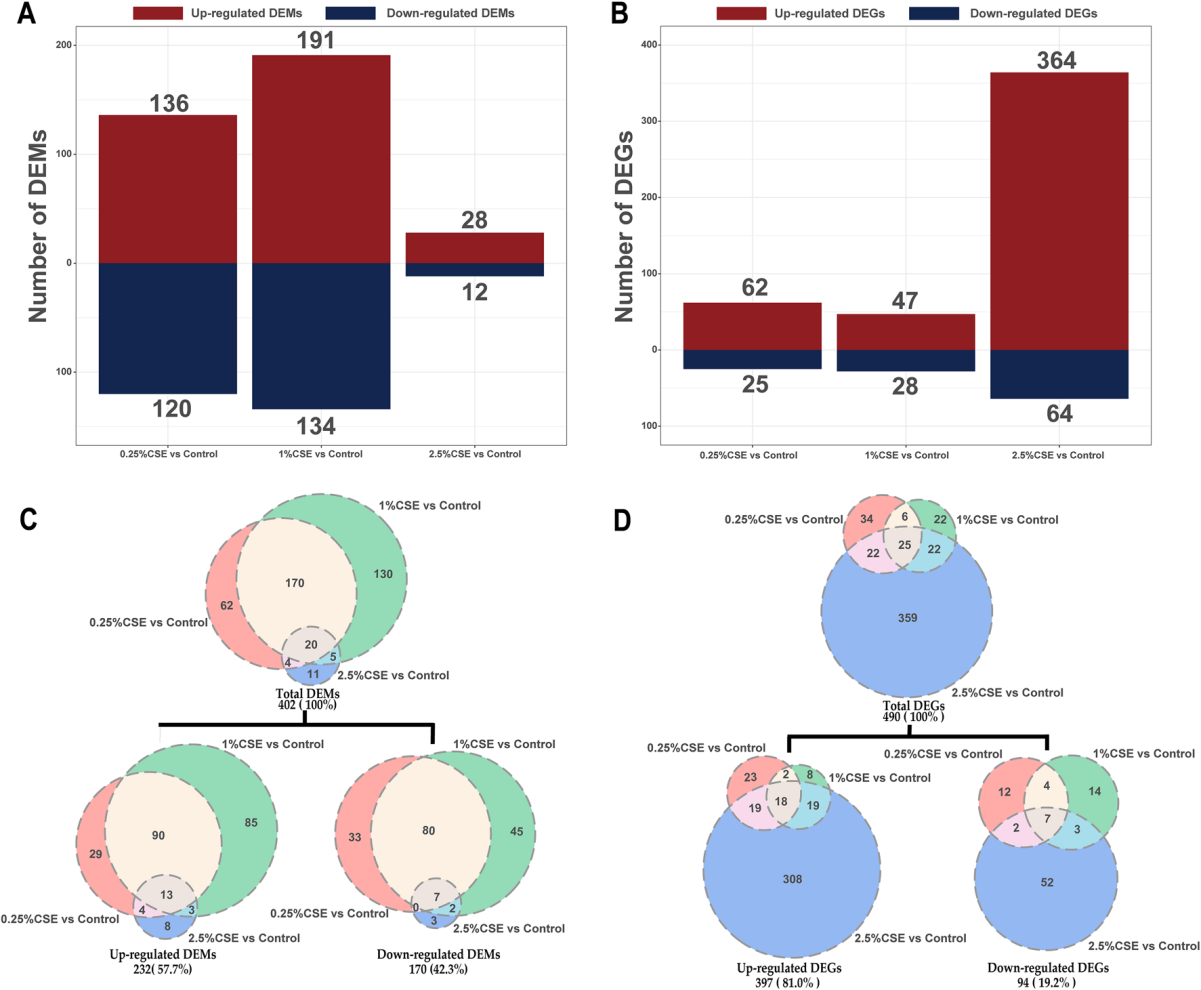
Effects of mRNA and miRNA transcriptome alterations by CSE exposure. The column diagram displaying the DEM and DEG counts (**A**, **B**). The overlaps of the DEM and DEG sets from the three comparison groups (0.25% CSE vs control, 1% CSE vs control, and 2.5% CSE vs control) shown in the Venn diagram (**C**, **D**). For a more thorough breakdown of DEMs and DEGs distribution, see Additional file 3: Fig. S2

The number of DEMs initially increased and then decreased as CSE concentration increased. However, the number of DEGs initially decreased and then increased (Fig. 2A, B). miRNA and mRNA transcription were more affected in the experimental groups than in the control group. Exposure to 2.5% CSE yielded a greater effect on gene expression than exposure to the other two concentrations, although 1% CSE exerted a greater effect on miRNA expression. In total, 402 DEMs were identified, among which 57.7% were upregulated and 42.3% were downregulated (Fig. 2C). Simultaneously, 490 DEGs were identified, among which 81.0% were upregulated and 19.2% were downregulated (Fig. 2D). The volcano plot (Additional file 3: Fig. S2) provides a visual representation of the miRNAs and mRNAs in each concentration group, highlighting the top 10 DEMs or DEGs with significant differences (Additional file 4: Fig. S3).

### Enrichment analysis of DEGs

**Fig. 3:**
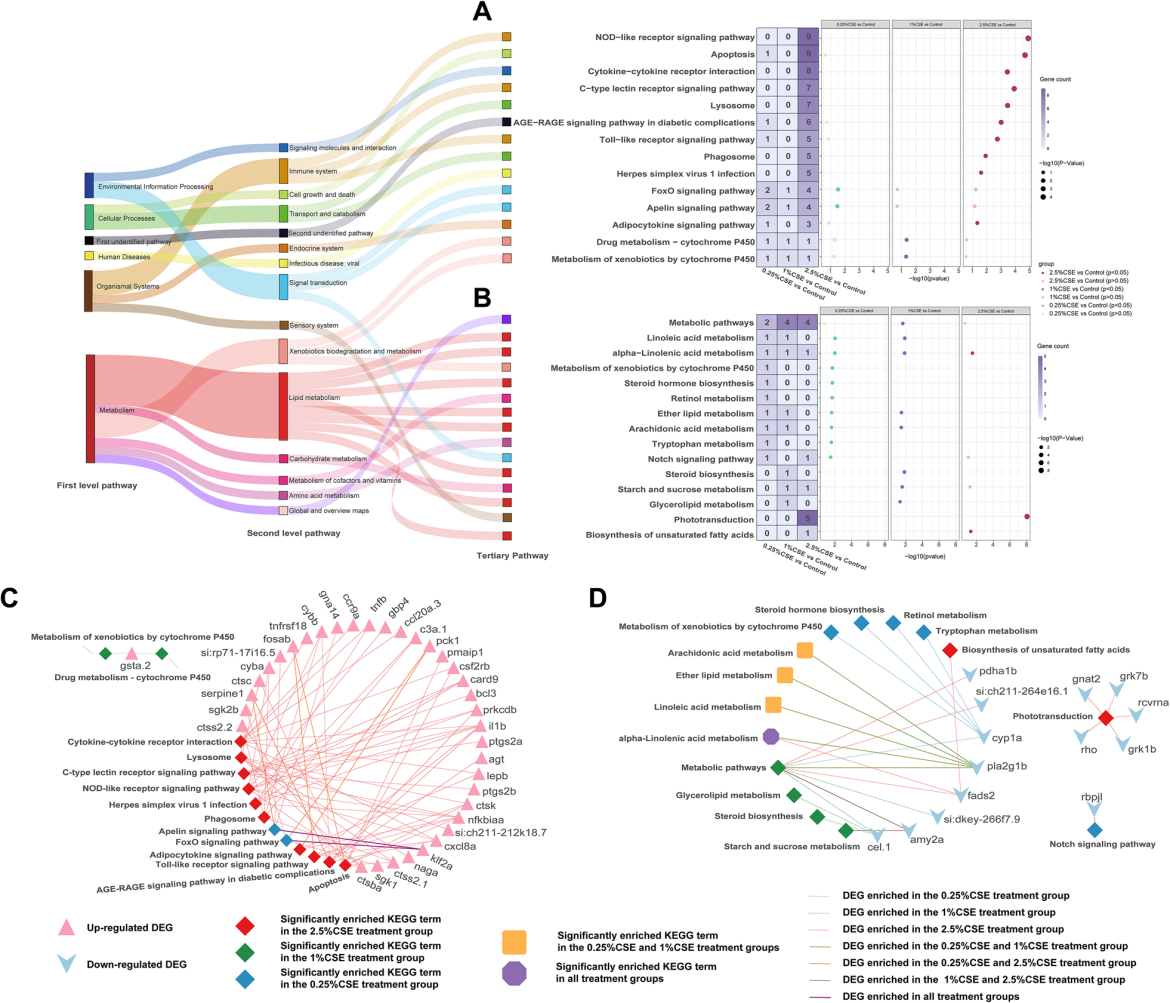
Effect of CSE exposure on KEGG functional enrichment in DEG group. Total significant KEGG pathways in DEGs with increased (**A**) and decreased expression (**B**) respectively. The tertiary pathways of the KEGG findings are classified in the left Sankey diagram. The quantity of DEGs enriched in the depicted pathways was shown on the purple Heat maps. Gene-term networks illustrating the connection between enriched DEGs and both up-regulated (**C**) and down-regulated (**D**) KEGG terms

To elucidate the mechanism underlying CSE-induced damage, we conducted a functional analysis of DEGs at various doses. KEGG enrichment analysis showed that the 2.5% CSE treatment group was enriched with more pathways for upregulated DEGs and fewer for downregulated DEGs than the other two groups. Specifically, the 2.5% CSE treatment group showed significant upregulation of pathways related to cellular processes and the immune system. Only two pathways—“Phototransduction” and “Biosynthesis of unsaturated fatty acids”—were downregulated. Lipid metabolism-related pathways were primarily downregulated in the 0.25 and 1% CSE treatment groups, which were similar. Notably, alpha-linolenic acid metabolism was involved in all three experimental groups (Fig. 3A, B). To further elucidate the primary functions of these pathways, we employed gene-KEG Gterm network graphs to demonstrate the complex relationships between terms in corresponding exposure to different concentrations of CSE and identified key genes associated with changes in CSE concentration (Fig. 3C, D).

**Fig. 4:**
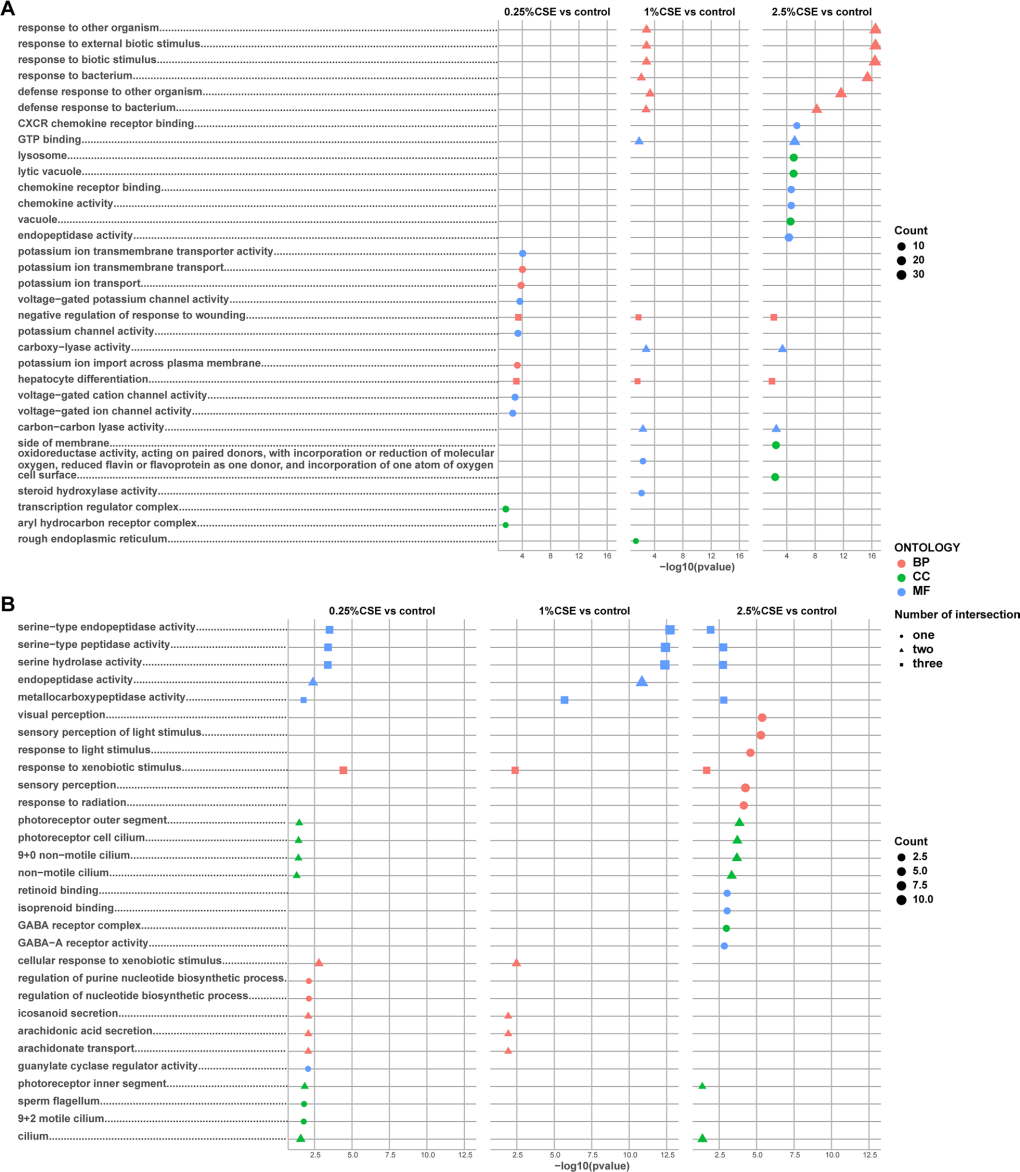
GO functional enrichment analysis in DEG group exposed by CSE. Top 5 GO terms of MF, CC, and BP categories with the greatest significance in up-regulated (**A**) and down-regulated (**B**) DEGs of three different concentration treatments exposed by CSE. *MF* Molecular Function, *CC* cellular component, and *BP* biological process

**Fig. 5:**
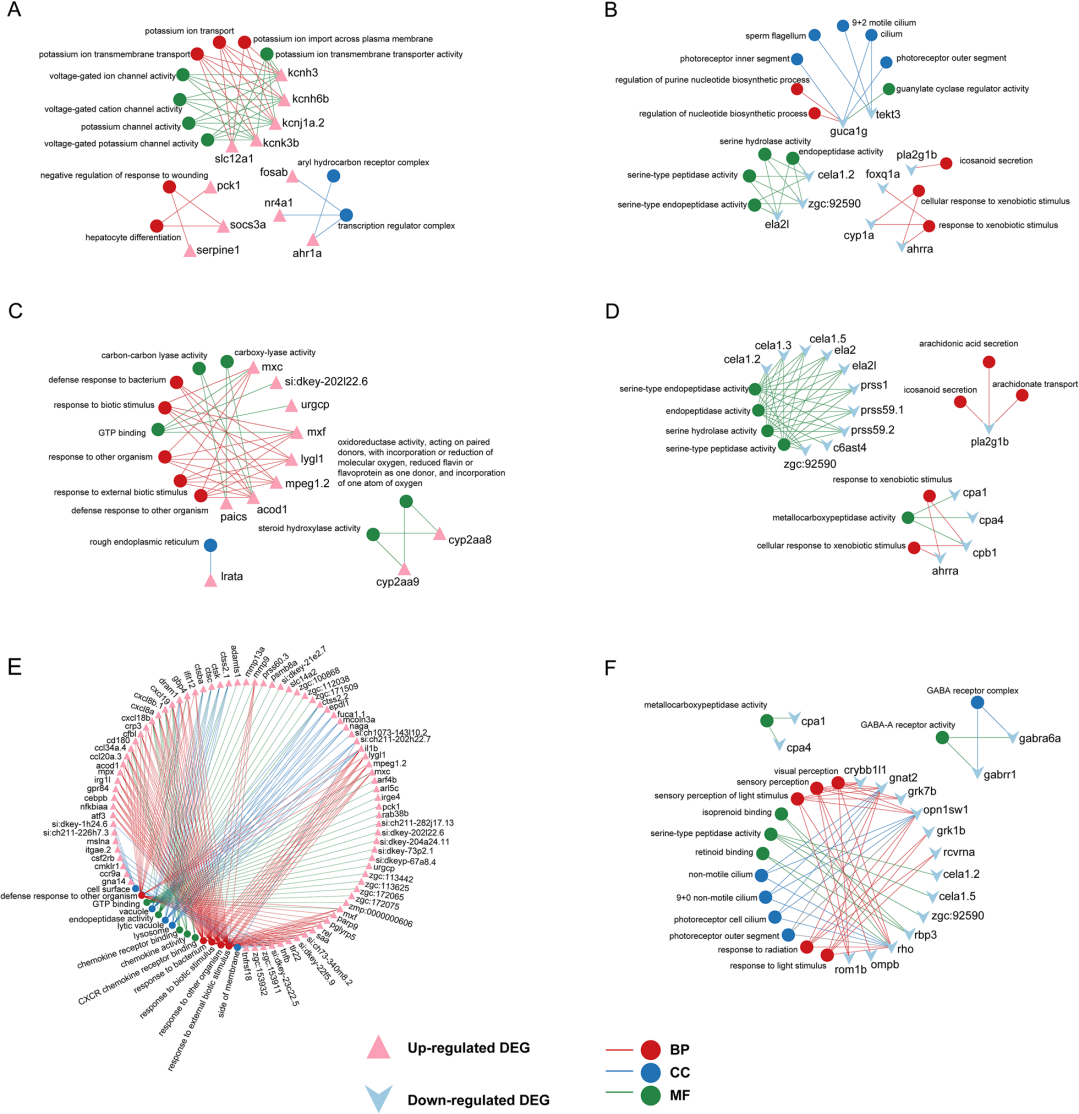
The relation between enriched GO term and involved gene in DEG group. Gene-term networks in up- and down-regulated GO terms in 0.25% CSE vs control (**A**, **B**), 1% CSE vs control (**C**, **D**), and 2.5% CSE vs control (**E**, **F**). GO terms are top 5 GO terms of MF, CC, and BP categories with the greatest significance in up-regulated and down-regulated DEGs of three treatment groups. *MF* Molecular Function, *CC* cellular component, and *BP* biological process

The GO results revealed notable similarities in the upregulated terms between the 1 and 2.5% CSE treatment groups. Meanwhile, the 0.25 and 1% CSE treatment groups exhibited clear parallelism in the downregulated terms (Fig. 4). Specifically, among the upregulated outcomes, processes and activities related to potassium ion transport were significantly enriched in the 0.25% CSE treatment group. Nevertheless, as the concentration increased, the 2.5% CSE treatment group was mostly associated with components involved in cell transport, namely, vesicle and lysosome. The 1 and 2.5% CSE treatment groups had comparable enrichment results in response to biological stimuli (Fig. 4A). In the downregulated outcomes, serine-related enzymes and metallocarboxypeptidase activity were commonly observed in all experimental groups. The 0.25% CSE treatment group primarily exhibited involvement in nucleotide synthesis, lipid transport, and photoreceptor components. Notably, lipid transport processes, such as secretion and transport of arachidonic acid, were also downregulated in the 1% CSE treatment group, and photoreceptor components were enriched in downregulated DEGs in the 2.5% CSE treatment group. Additionally, downregulated visual conduction was particularly evident in the 2.5% CSE treatment group (Fig. 4B). Similarly, we constructed gene-GO term network diagrams that visually demonstrated how certain genes, through their regulatory functions, establish connections between distinct biological processes, cellular components, and molecular functions (Fig. 5). This visual representation offers a comprehensive overview of the complex interactions influenced by CSE treatment at different concentrations.

**Fig. 6:**
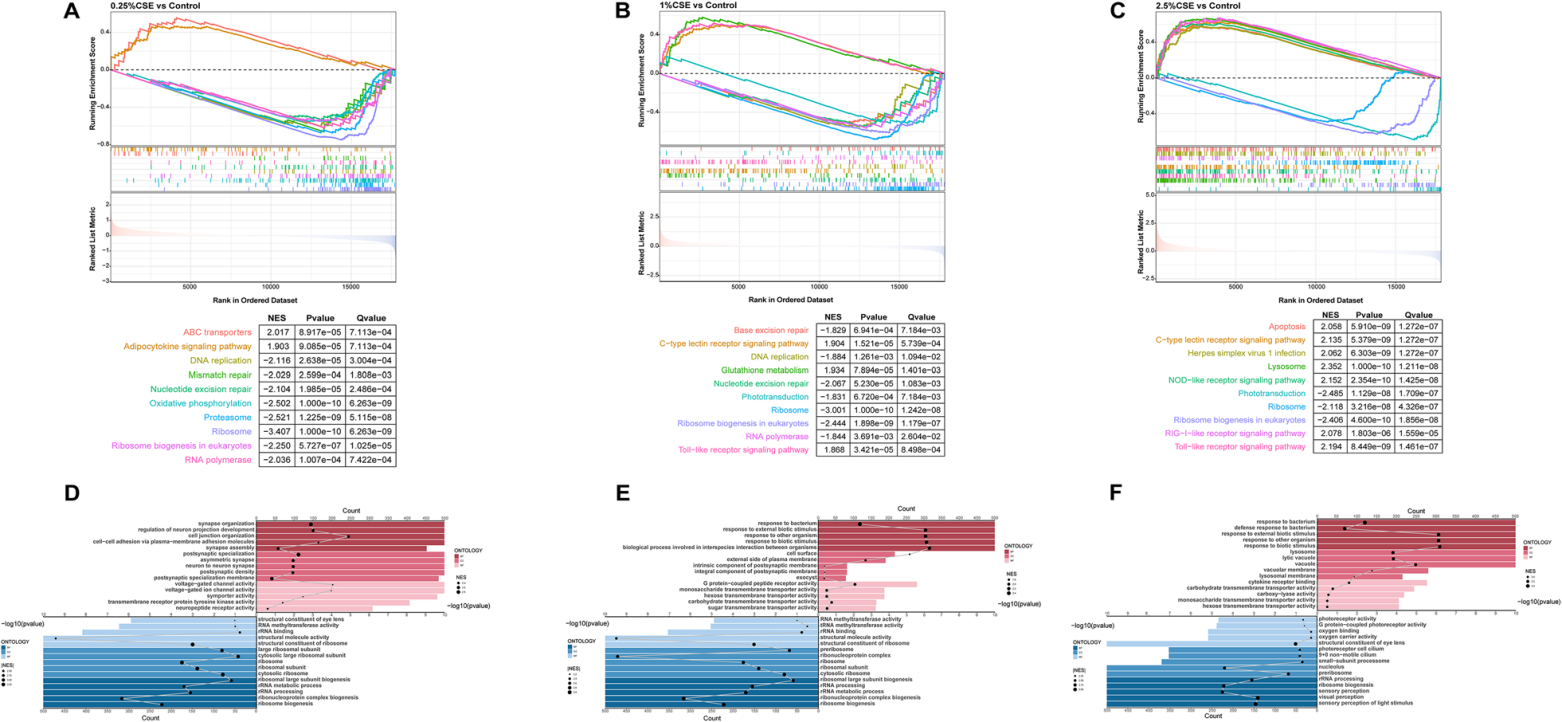
The GSEA results enriched by three gene datasets from various concentration treatments treated by CSE. Top 10 KEGG terms with the highest NES in 0.25% CSE vs control (**A**), 1% CSE vs control (**B**), and 2.5% CSE vs control (**C**). Top 5 GO terms of the MF, CC, and BP categories with the largest NES in 0.25% CSE vs control (**D**), 1% CSE vs control (**E**), and 2.5% CSE vs control (**F**).The horizontal bar length corresponds to the magnitude of the p-value, while the lines show changes in the enriched gene count

To further explore the underlying mechanism through which CSE affects coordinated embryonic development, we performed GSEA for all genes. Downregulated pathways related to DNA damage repair were significant in all treatment groups, further supporting the KEGG and GO functional enrichment results (Fig. 6). The upregulated GSEA results were almost consistent with the upregulated functional enrichment results, such as lysosome and apoptosis in the 2.5% CSE treatment group.

### Enrichment analysis of DEM target genes

**Fig. 7:**
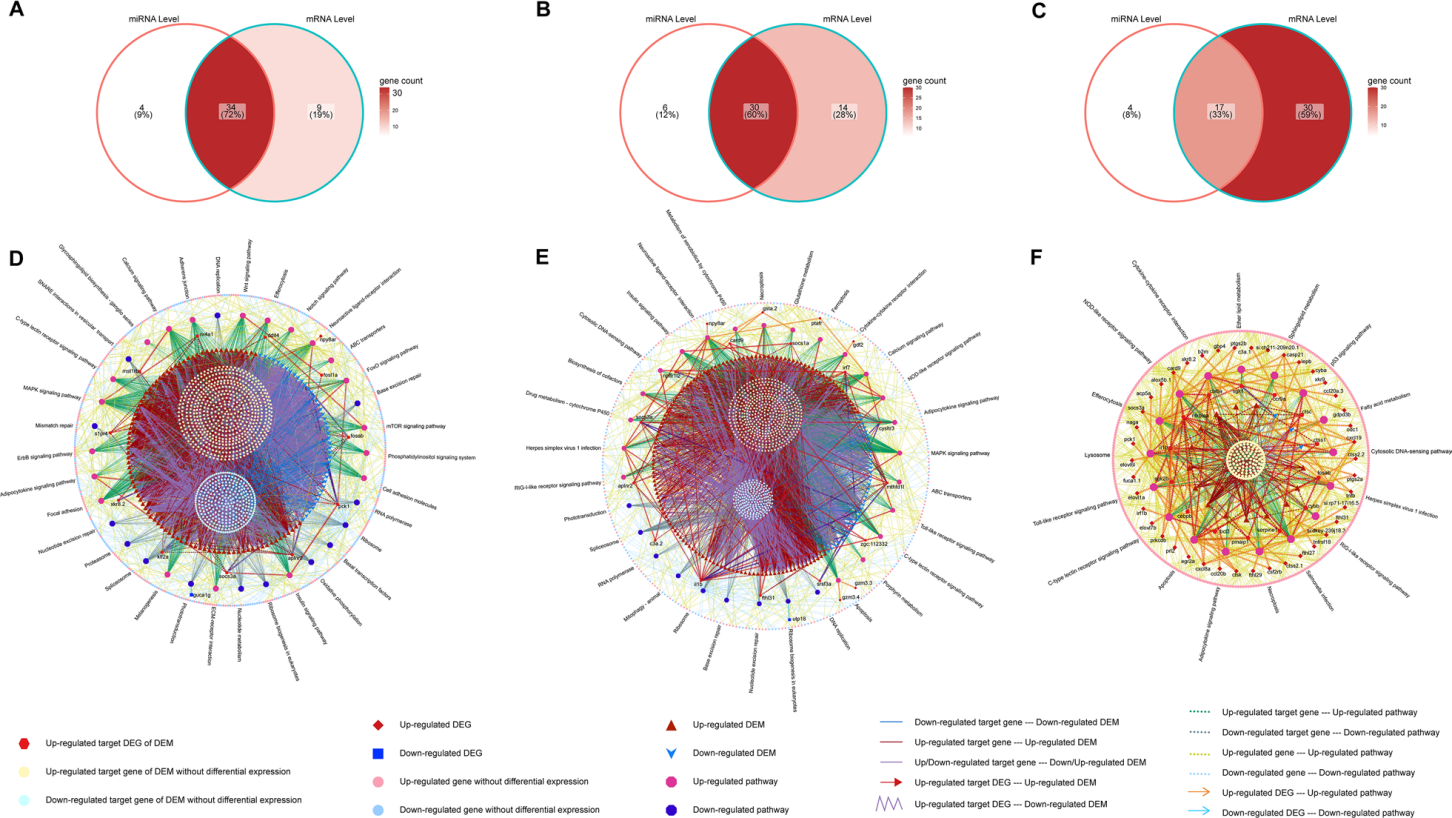
GSEA-KEGG terms interconnected between miRNA and mRNA levels. Venn diagram of KEGG terms in 0.25%CSE vs control (**A**), 1%CSE vs control (**B**), and 2.5%CSE vs control (**C**). The miRNA–mRNA–pathway networks for 0.25%CSE vs control (**D**), 1%CSE vs control (**E**), and 2.5%CSE vs control (**F**)

**Fig. 8:**
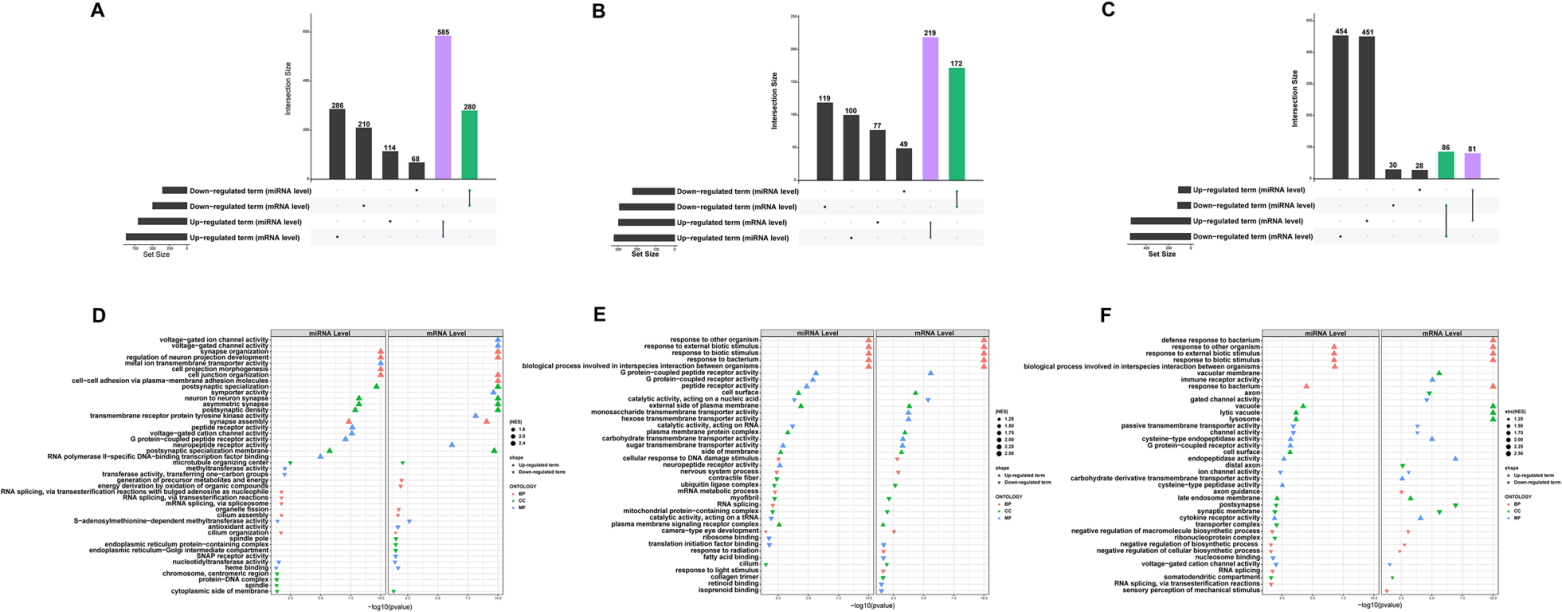
GSEA-GO terms interconnected between miRNA and mRNA levels. Intersected GO terms between miRNA and mRNA levels in 0.25% CSE vs control (**A**), 1% CSE vs control (**B**), and 2.5% CSE vs control (**C**). The upset plot mainly focused on the crossroads scenario. The specifics of the intersecting GO terms were presented in dot plots (**D**–**F**). *MF* Molecular Function, *CC* cellular component, and *BP* biological process

We initially identified the DEM target genes and subsequently conducted GSEA analyses of the genes to evaluate the association between responsive miRNAs and mRNAs. To identify the most valuable pathways, we first used Venn diagrams to illustrate the overlap between the significantly enriched GSEA-KEGG results in the miRNA and mRNA groups (Fig. 7A–C). These significant intersected KEGG terms were elucidated using miRNA-mRNA-pathway regulatory networks. The number of significantly overlapped pathways, DEMs, and their target genes decreased as the concentration of CSE exposure increased, and the number of DEGs increased. Furthermore, in the experimental groups, eventually, there was an increase in the prevalence of upregulated pathways, but the downregulated pathways, largely associated with DNA repair, decreased with an increase in CSE concentration. Notably, apoptosis and lysosome were two pathways significantly upregulated throughout the bioinformatics analysis. However, the lipid metabolism manner was only significantly activated in the 2.5% CSE concentration group in this case; by contrast, in our prior bioinformatics study, it was strongly correlated with the 0.25 and 1% CSE concentration groups (Fig. 7D–F). The noteworthy GO results from the intersection are equally remarkable. As illustrated by the upset plot, the number of GO terms at the intersection gradually decreased with concentration increased (Fig. 8A–C). Furthermore, at the miRNA and mRNA levels, the important pathways with high NES rankings remained largely consistent (Fig. 8D–F). The significant enrichment outcomes obtained from the intersection at both the miRNA and mRNA levels hold paramount importance.

### Validation of significant DEG expression using RT-qPCR

**Fig. 9:**
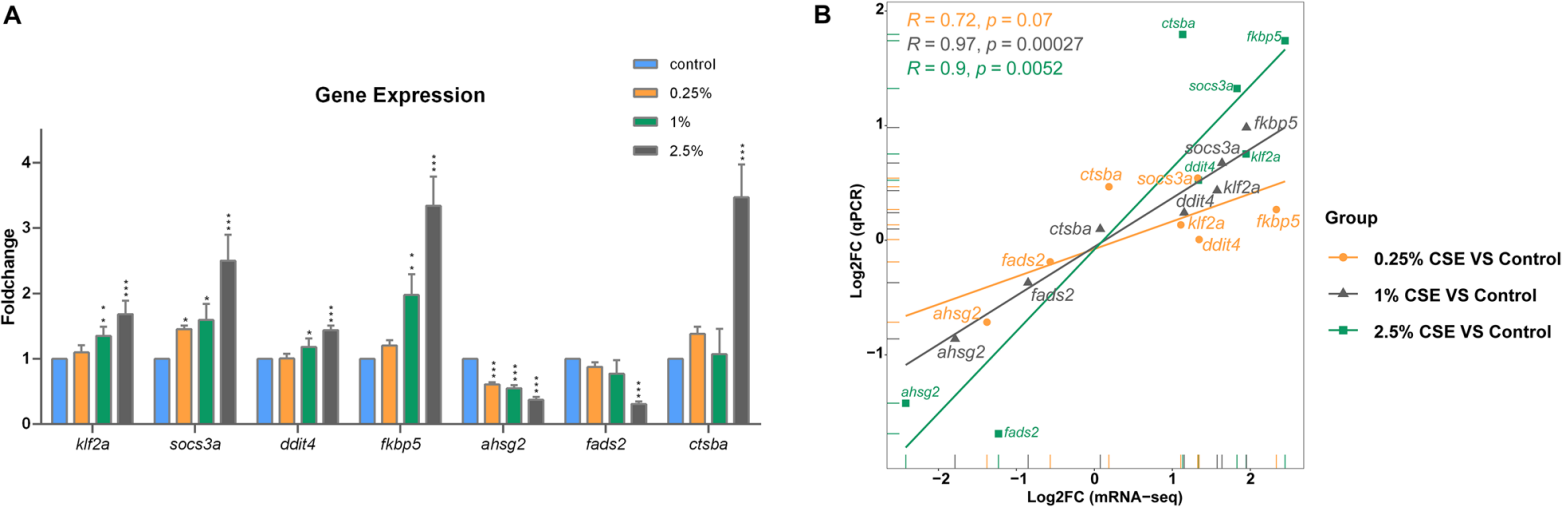
RT-qPCR verification of the chosen DEGs' expression. The bar graph with various groups showing the relative levels of gene expression (**A**). The linear regression figure depicting the DEGs’ expression consistency between qPCR and sequencing data (**B**)

The RT-qPCR validation analysis revealed that the expression levels of *klf2a*, *socs3a*, *ddit4*, *fkbp5, ahsg2*, *fads2*, and *ctsba* were consistent with the changes observed in the mRNA-seq transcriptome across the three concentration groups (Fig. 9A). Additionally, the expression levels of the selected genes, determined through qPCR, had a significant positive correlation with the expression levels of genes obtained from the mRNA-seq data across all three CSE concentration groups (0.25% CSE group: R = 0.72, p = 0.07; 1% CSE group: R = 0.97, p = 0.00027; 2.5% CSE group: R = 0.9, p = 0.0052) (Fig. 9B).

## Discussion

As environmentally persistent and inevitable pollutants, tobacco smoke and its residues have been proven to be significant environmental contaminants, posing potential health hazards for pregnant women and their unborn fetuses [3, 7]. However, the precise mechanism through which tobacco pollutants affect embryonic development has not been fully elucidated. Therefore, this study aimed to elucidate the possible underlying mechanisms of CSE-induced developmental toxicity in zebrafish embryos by combining embryonic development observations and bioinformatics analysis results.

The embryonic toxicity induced by CSE manifested in deteriorating developmental indicators of zebrafish larvae, including increased rates of deformities, elevated mortality, and exacerbated cellular apoptosis. This dose–response trend in toxic effect has been consistently observed in other animal models, suggesting a potentially universal impact of CSE on embryonic toxicity [28–30]. Furthermore, utilizing AO staining techniques, notable morphological abnormalities and excessive cellular apoptosis had been observed in the head and cardiac regions of zebrafish, shedding light not only on the primary toxic effects of CSE exposure on embryos but also hinting at the head and heart as potential key target organs. However, solely examining the developmental outcomes of animals exposed to ingredients of CS, as these studies have done, is insufficient to elucidate the complex mechanisms underlying CSE-induced embryonic developmental toxicity [17, 31, 32]. To improve the understanding of these mechanisms, we assessed the dysregulation related to genetic damage repair, apoptosis, and lipid metabolism, integrating analyses across various transcriptional levels and different concentrations of CSE exposure.

Many harmful components in CSE, including nicotine and benzo[a]pyrene, directly target cellular DNA, leading to genetic toxicity [33–35]. While our study did not directly observe damage to embryonic genetic material, a comprehensive analysis of GSEA results across all treatment groups unveiled various downregulations in DNA repair processes at both miRNA and mRNA transcriptional levels. These downregulations included critical mechanisms such as base excision repair, mismatch repair, and nucleotide excision repair, consistent with studies indicating that components of CS inhibit DNA repair [35–37]. For instance, Cui et al. demonstrated that heightened oxidative stress induced by CS diminishes base excision repair capacity, consequently exacerbating DNA damage [38]. In response to compromised genetic material, cells initiate a series of repair processes to shield themselves from further harm [36, 37, 39]. However, impediments in DNA repair processes can profoundly affect cellular viability and genomic stability [37, 39]. Given that normal embryonic development heavily relies on precise genetic regulation [40, 41], it is plausible that CSE not only directly damages genetic material but also compromises the organism's DNA repair capacity, potentially resulting in embryonic growth delays, deformities, or death. Furthermore, in line with the findings of Park et al., our GSEA analysis revealed dynamic alterations in RNA metabolism processes, such as ribosome biogenesis, transcription, and translation, which mirror those of DNA damage repair in our research [42]. Due to the crucial role of RNA metabolism in DNA damage repair, any impairment in RNA metabolism could significantly reduce the effectiveness of restoring damaged genetic material [43, 44]. These findings shed light on the complex interactions of CSE with genetic material damage and repair mechanisms, emphasizing the significance of genotoxicity as a crucial component of CSE-induced developmental process.

CSE significantly increases cellular death, leading to cytotoxicity that adversely affects embryonic developmental [45, 46]. This correlation was confirmed through apoptosis detection and enrichment analysis in our study, highlighting the direct correlation between rising CSE concentrations and heightened apoptosis levels [47–49]. Apoptosis, essential for eliminating damaged cells and supporting normal development, is intricately linked to early embryonic development, emphasizing the importance of precise cell death regulation [50]. Studies on toxicant-induced developmental defects have underscored excessive embryonic cell death as a pivotal precursor to structural abnormalities [51]. Through comprehensive analysis, a cluster of genes closely associated with apoptosis, including *ctsba*, *ctsc*, and *ctsk*, primarily encoding cathepsin [52–54]. These genes notably involved in essential components of autophagy process, such as lysosomes, vacuoles, and lytic vacuoles in our research. Apoptosis and autophagy are indispensable cellular death mechanisms crucial for embryonic development [55]. While apoptosis eliminates damaged cells, autophagy degrades apoptotic remnants, collectively maintaining cellular balance and facilitating tissue remodeling [56]. Dysfunctions in autophagy may lead to delayed embryonic development, anomalies, or premature demise [57]. These genes may thus have far-reaching effects on embryonic development by regulating autophagy and apoptosis processes. Additionally, our study unveils that *ctsba* acts not only as a target gene for DEMs but also emerges as a DEG itself. Cathepsin B encoded by the *ctsba* gene, predominantly localized within lysosomes, plays a pivotal role in apoptosis and autophagy [52, 58]. Cathepsin B serves a dual role in autophagy, actively promoting the apoptosis process [58]. Components in CS can significantly raise cathepsin B levels at both the RNA and protein levels, consistent with our findings [59, 60]. Moreover, the literature has shown that embryos of poor quality exhibit elevated cathepsin B activity and mRNA expression compared to those of good quality, and that inhibiting cathepsin B an lead to reduce apoptosis levels and improve embryonic development potential [61–63]. Additionally, Yvette et al. have definitively established the critical role of *ctsba* in morphogenesis and that the absence of this gene leads to the abnormal split-top embryos [64]. Taken together, these findings underscore the intricate and pivotal roles of CSE-induced embryonic developmental cytotoxicity, the *ctsba* gene, and its encoded cathepsin B in regulating apoptosis and autophagy during embryonic development.

Recent research has shed light on the interference of toxicant exposure with lipid metabolism and its deleterious consequences on embryo development [65, 66]. Although direct evidence remains elusive, the KEGG enrichment analysis of DEGs in different concentration groups revealed a notable downregulation of pathways associated with lipid metabolism, suggesting potential impairment in lipid metabolism among embryos exposed to CSE. Lipids supply energy and generate signaling molecules such as lysophosphatidic acid and prostaglandins for embryonic development; however, abnormalities in lipid content and metabolites can cause dysplasias and defects [67, 68]. Remarkably, gene-KEGG term network diagrams unveil an intriguing pattern: the involved genes shift from *pla2g1b* to *fads2* with increasing CSE concentration, despite alpha-Linolenic acid metabolism being enriched in all treatment groups. This discovery offers a new viewpoint, highlighting the crucial role of fads2 in CSE-induced metabolic harm during embryogenesis. The *fads2* gene encodes delta-6 desaturase, uniquely capable of synthesizing long-chain polyunsaturated fatty acids (LC-PUFAs) in zebrafish [69, 70]. LC-PUFAs, which are crucial for embryonic growth, not only serve as essential lipid constituents but also play vital roles in cell membrane composition and organismal signal transduction [71, 72]. Consequently, the downregulation of *fads2* induced by CSE may disrupt lipid metabolism, resulting in a significant reduction in LC-PUFAs. This disturbance may hinder embryos from developing normally, resulting in morphological abnormalities and detrimental effects on tissue differentiation [73–75]. In summary, this study elucidates the metabolic toxicity of CSE on embryonic development and underscores the critical involvement of *fads2* in lipid metabolism.

Treating embryos with a CSE containing multiple components could enhance the understanding of the mechanisms underlying the harmful effects of tobacco contaminants [17]. In this study, a combination of morphological and bioinformatics techniques was employed to investigate the impact of CSE on early embryonic development from various perspectives. Our findings demonstrated genetic toxicity, cytotoxicity, and metabolic toxicity, respectively, caused by the damaged genetic material, disordered apoptosis, and lipid metabolism disorders in embryos exposed to CSE. This research contributes novel toxicological data, thereby advancing the comprehensive understanding of CSE-induced developmental toxicity; however, further experimental validation is necessary.

## Conclusions

In conclusion, our extensive investigation has demonstrated that the exposure of zebrafish embryos to CSE resulted in developmental toxicity, particularly affecting the head and heart. Through the comprehensive analysis of miRNA and mRNA transcriptomics, we have discovered a close correlation between embryonic developmental toxicity and compromised genetic material damage repair, deregulated apoptosis, and disturbed lipid metabolism. Furthermore, our findings suggest that the *ctsba* and *fads2* genes may play pivotal roles in regulating apoptosis-autophagy and lipid metabolism, respectively. These findings have provided valuable insights into the potential mechanisms by which CSE induces embryonic developmental toxicity in zebrafish.

### Supplementary Information

**Additional file 1: Table S1.** The primer sequences used for gene expression studies were shown in Table S1.

**Additional file 2: Figure S1.** Typical malformations of zebrafsh larvae exposed to various concentrations of CSE (0, 0.25%, 1% and 2.5%) for 7dpf (A) and 96 hpf (B).

**Additional file 3: Figure S2.** Volcanic maps of DEMs (A, B, C) and DEGs (D, E, F) for three different comparison groups (0.25% CSE vs control, 1% CSE vs control, and 2.5% CSE vs control).

**Additional file 4: Figure S3.** Thorough investigation of the PPI network submodule. The PPI network submodule with the highest MCODE score in 0.25% CSE vs control (A), 1% CSE vs control (B), and 2.5% CSE vs control (C). Hub DEGs are represented by the nodes, and node interactions are represented by the edges. The log2FC value caused the nodules' color to alter. With the aggregation fraction, the margins' color and thickness altered. Total significant KEGG pathways of both elevated and downregulated DEGs and their gene-term network in three distinct concentration treatments (D, E). Top 10 GO terms with the most significance obtained by upregulated and downregulated DEGs and their gene-term network in three different concentration treatments exposed by CSE (F, G, H, I, J, K). MF: Molecular Function, CC: cellular component, and BP: biological process.

## Data Availability

The original contributions presented in the study are included in the article/Supplementary Material. Further inquiries can be directed to the corresponding authors.

## Abbreviations

CSE: Cigarette smoke extract
CS: Cigarette smoke
miRNA: MicroRNA
mRNA: Messenger RNA
RT-qPCR: Quantitative real-time PCR
Hpf: Hour post-fertilization
Dpf: Day post-fertilization
AO staining: Acridine orange staining
Padj: Adjusted p-value
DEGs: Differentially expressed genes
DEMs: Differentially expressed miRNAs
KEGG: Kyoto Encyclopedia of Genes and Genomes
GO: Gene Ontology
GSEA: Gene Set Enrichment Analysis
LC-PUFAs: Long-chain polyunsaturated fatty acids
