# Supplementary material for: Integrated mRNA- and miRNA-sequencing analyses unveil the underlying mechanism of tobacco pollutant-induced developmental toxicity in zebrafish embryos
Source: J Transl Med. 2024 Mar 8;22:253. doi: 10.1186/s12967-024-05050-9 (PMC10924323; doi:10.1186/s12967-024-05050-9)
Supplement: Supplementary file 1 — Additional file 1: Table S1. The primer sequences used for gene expression studies were shown in Table S1. [file 12967_2024_5050_MOESM1_ESM.docx]

| **NO.** | **Gene Name** | **Primer Sequences(5’-3’)** |
| --- | --- | --- |
| **1** | ***klf2a*** | F-AGAATAACAGACGACGAAGA  R-TCAGGCACCGAGTATGTA |
| **2** | ***socs3a*** | F-ACTGGTCCAGCACTACATGC  R-CAGGCCCAAAAACGTAAGCC |
| **3** | ***ddit4*** | F-TCGACCCTTACCTAGTGCCA  R-CACACAAACGGAGGAGACCA |
| **4** | ***fkbp5*** | F-ATGGTGGGATCGTGAGGAGA  R-AAAGGTGACATCCCGCGAAT |
| **5** | ***ahsg2*** | F-TCAGATTGTCAGTGGAGTG  R-GGATAGCAACAGTGTGATTC |
| **6** | ***ctsba*** | F-GCTGTCGTCCATATACCATTGAAC  R-TGTCTCCACCCTCTCCTGAAC |
| **7** | ***fads2*** | F-GACAGCAGACAGACCGAATCAC  R-AACCTTCCTCTCCACCACCAC |
| **8** | ***bcl2a*** | F-AACCGACTCTTTCCTGCTCG  R-TTCAGAGTTGTTCCCTCCGC |
| **9** | ***bax*** | F-GGCTATTTCAACCAGGGTTCC  R-TGCGAATCACCAATGCTGT |
| **10** | ***eef1a1l1*** | F-TACCCTCCTCTTGGTCGCTT  R-GAGGTTGGGAAGAACACGCC |

**The primer sequences used for gene expression studies were shown as follow:**
