# Supplementary figures and images for: Integrated mRNA- and miRNA-sequencing analyses unveil the underlying mechanism of tobacco pollutant-induced developmental toxicity in zebrafish embryos
Source: J Transl Med. 2024 Mar 8;22:253. doi: 10.1186/s12967-024-05050-9 (PMC10924323; doi:10.1186/s12967-024-05050-9)

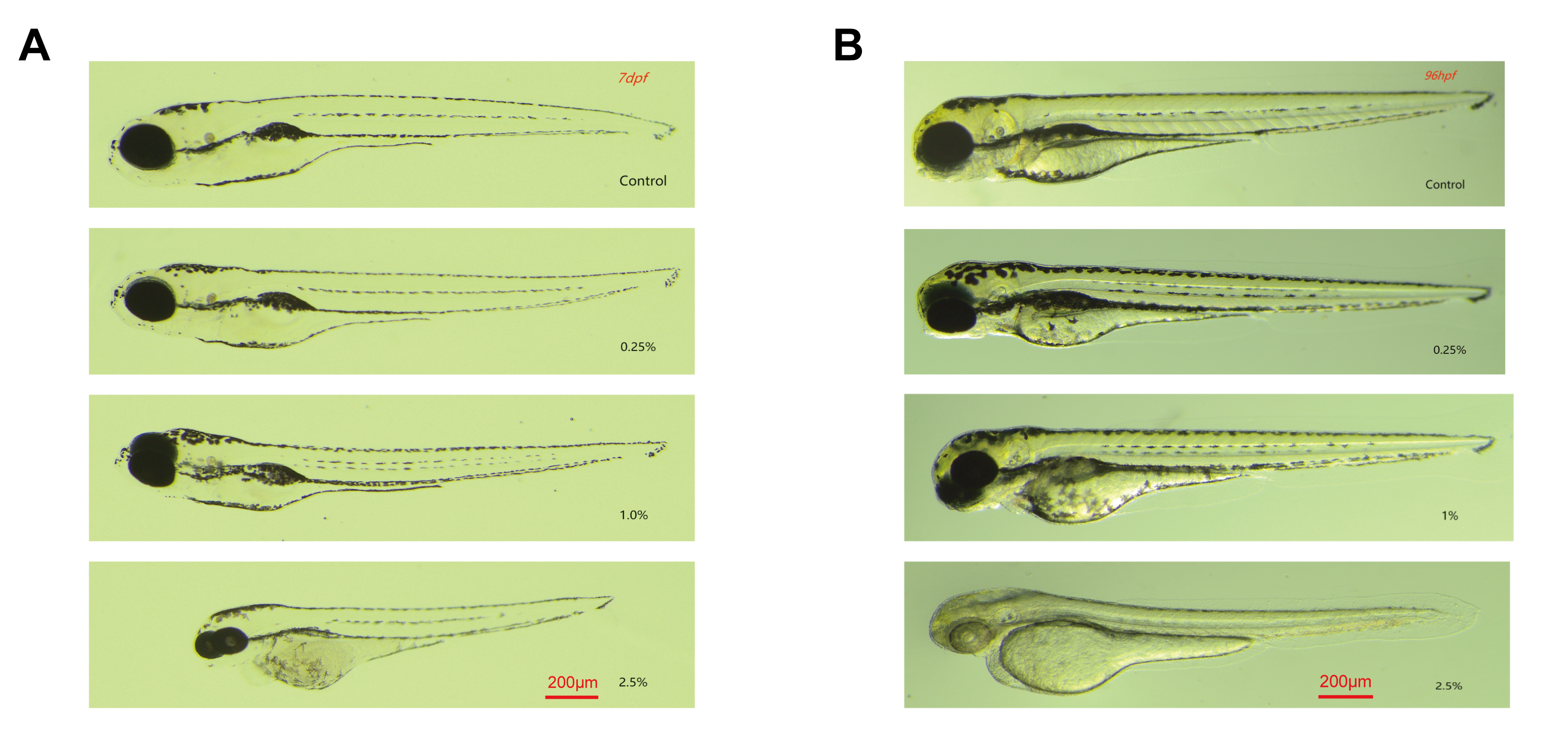

Supplement: Supplementary file 2 — Additional file 2: Figure S1. Typical malformations of zebrafsh larvae exposed to various concentrations of CSE (0, 0.25%, 1% and 2.5%) for 7dpf (A) and 96 hpf (B). [file 12967_2024_5050_MOESM2_ESM.tif]

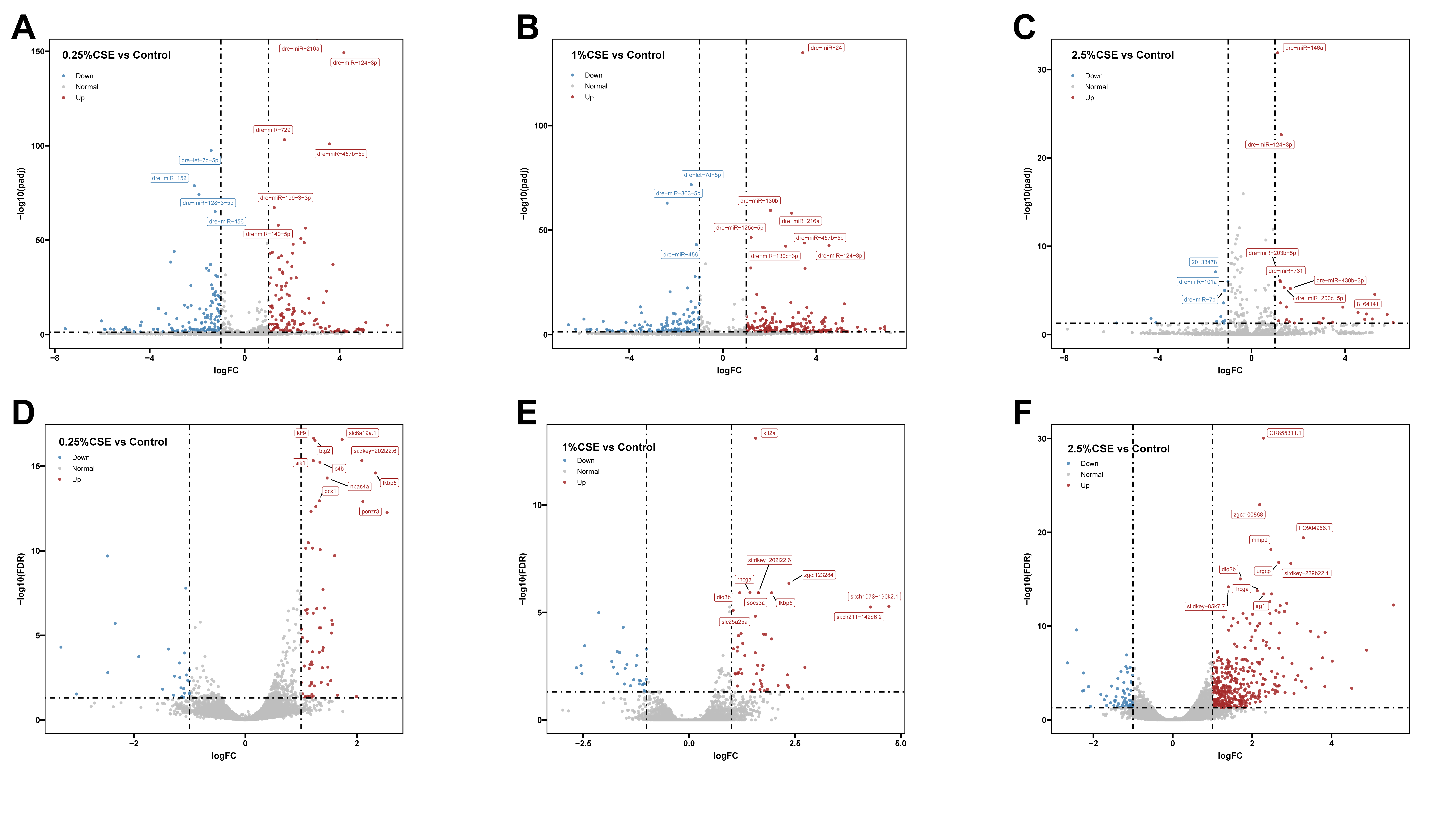

Supplement: Supplementary file 3 — Additional file 3: Figure S2. Volcanic maps of DEMs (A, B, C) and DEGs (D, E, F) for three different comparison groups (0.25% CSE vs control, 1% CSE vs control, and 2.5% CSE vs control). [file 12967_2024_5050_MOESM3_ESM.tif]
